# How healthy and affordable are foods and beverages sold in school canteens? A cross-sectional study comparing menus from Victorian primary schools

**DOI:** 10.1017/S136898002300126X

**Published:** 2023-11

**Authors:** Amy Hill, Miranda Blake, Laura Veronica Alston, Melanie S Nichols, Colin Bell, Penny Fraser, Ha ND Le, Claudia Strugnell, Steven Allender, Kristy A Bolton

**Affiliations:** 1 Global Centre for Preventive Health and Nutrition (GLOBE), Institute for Health Transformation, Deakin University, Geelong 3220, VIC, Australia; 2 Deakin Rural Health, Faculty of Health, Deakin University, Geelong 3220, VIC, Australia; 3 Research Unit, Colac Area Health, Colac 3250, VIC, Australia; 4 GLOBE, Institute for Health Transformation, School of Medicine, Deakin University, Geelong 3220, VIC, Australia; 5 Institute for Physical Activity and Nutrition, Deakin University, Geelong 3220, VIC, Australia

**Keywords:** Nutrition, Canteen menus, Pricing, School, Children, Policy

## Abstract

**Objective:**

Government policy guidance in Victoria, Australia, encourages schools to provide affordable, healthy foods in canteens. This study analysed the healthiness and price of items available in canteens in Victorian primary schools and associations with school characteristics.

**Design:**

Dietitians classified menu items (main, snack and beverage) using the red, amber and green traffic light system defined in the Victorian government’s School Canteens and Other School Food Services Policy. This system also included a black category for confectionary and high sugar content soft drinks which should not be supplied. Descriptive statistics and regressions were used to analyse differences in the healthiness and price of main meals, snacks and beverages offered, according to school remoteness, sector (government and Catholic/independent) size, and socio-economic position.

**Setting:**

State of Victoria, Australia

**Participants:**

A convenience sample of canteen menus drawn from three previous obesity prevention studies in forty-eight primary schools between 2016 and 2019.

**Results:**

On average, school canteen menus were 21 % ‘green’ (most healthy – everyday), 53 % ‘amber’ (select carefully), 25 % ‘red’ (occasional) and 2 % ‘black’ (banned) items, demonstrating low adherence with government guidelines. ‘Black’ items were more common in schools in regional population centres. ‘Red’ main meal items were cheaper than ‘green’% (mean difference –$0·48 (95 % CI –0·85, –0·10)) and ‘amber’ –$0·91 (–1·27, –0·57)) main meal items. In about 50 % of schools, the mean price of ‘red’ main meal, beverages and snack items were cheaper than ‘green’ items, or no ‘green’ alternative items were offered.

**Conclusion:**

In this sample of Victorian canteen menus, there was no evidence of associations of healthiness and pricing by school characteristics except for regional centres having the highest proportion of ‘black’ (banned) items compared with all other remoteness categories examined. There was low adherence with state canteen menu guidelines. Many schools offered a high proportion of ‘red’ food options and ‘black’ (banned) options, particularly in regional centres. Unhealthier options were cheaper than healthy options. More needs to be done to bring Victorian primary school canteen menus in line with guidelines.

Schools are an important setting for establishing healthy dietary patterns and reducing risks of chronic disease like childhood overweight and obesity^(1,2)^. Unlike the USA, the UK and Japan where food is routinely provided for students at school, in Australia, food is typically prepared at home and taken to school or alternatively purchased from a school-based canteen or ‘lunch order’ system whereby parents can place orders for food which is either prepared by the canteen itself or from an offsite food provision service. Access to canteens and lunch orders is determined individually by the school and can range from access once or twice a week to every day. Historically, school canteens or lunch orders have offered a high proportion of energy-dense and less healthy foods such as pastries, cakes and sugar-sweetened beverages^(3,4)^, even though it is recognised that school food is important for health messaging and education^(4)^. Further, energy consumed from discretionary foods obtained at school, along with frequency of purchasing lunch at school canteens, has been associated with overweight and obesity^(5,6)^. The system of food provision in Australian schools has meant that health promotion efforts have focused both on parent and caregiver education and on policies designed to influence the availability and affordability of foods and beverages provided by school canteens.

Schools are a key setting for action in Australia’s recent National Obesity Strategy^(7)^. However to date, the presence of school nutrition policy has not necessarily translated to a healthy canteen^(8)^. Australia’s states and territories, which have responsibility for the education system, have progressively introduced policy guidelines for school food provision since 2005^(9)^. The Victorian state government’s Department of Education and Training first released a food service policy for schools in 2006, with guidelines on the provision of healthy food and drink underpinned by the Australian Dietary Guidelines^(10)^. Following this, the School Confectionary Guidelines were instituted in 2009^(11)^, and more recently, in June 2020, the policy name was simplified to ‘Canteens, Healthy Eating and Other Food Services’ policy with no change to policy content^(12)^. The policy uses a ‘traffic light’ classification system to guide food provision, with the availability, promotion, competitive pricing, and display of ‘Everyday’ food and drinks (‘green’ classification) encouraged^(12)^. ‘Green’ menu items should make up the majority (> 50 %) of available items. ‘Select Carefully’ (‘amber’ classification) food and drinks should be restricted and make up less than half of the canteen menu, while ‘Occasionally’ food and drink (‘red’ classification) should not be included on the regular menu^(12)^. Banned items on the policy include high sugar content drinks and confectionary (‘black’ classification)^(12)^. It is important to note that these guidelines are not mandated in government or other types of schools (e.g. Catholic/independent), nor formally monitored; however, schools are strongly encouraged to support a whole-school approach to health eating^(12)^.

Historically, studies have found poor adherence to school food policy, both Australia-wide^(13,14)^ and in Victoria specifically^(8,15)^. For example, an analysis of 106 school canteens in 2008 and 2009 found that on average only 20 % of items on the menus were ‘green’^(8)^. Furthermore, a recent analysis of healthy *v*. less healthy menu food options in school canteens revealed that less healthy food options were significantly cheaper^(14)^, though this study did not include drinks. A national analysis of school canteens^ ^13^^ found similar findings (albeit in secondary schools), with healthier items significantly more expensive than less healthy, and almost all schools offering red items contrary to advice provided in region-specific policies and guidelines^(13)^. Drink choices were dominated by red and amber options^(13)^. A recent cross-sectional study found that 38 % of schools reported that they had a healthy canteen policy, indicating interest from schools to adopt healthy practices^(16)^. However, the implementation of that policy and whether it translated to healthier canteens was not evaluated^(16)^.

Diet-related chronic disease is higher in rural Australia compared with urban counterparts, and improving the healthiness of diet is a key priority^(17)^. Furthermore, higher rates of childhood obesity (a key risk factor for chronic diseases) in Victorian regional areas have also been reported (32 % of girls and 29 % boys outside of major cities^(18)^ compared with national average of about 25 %^(19)^. Therefore, it is important to explore differences in the healthiness of canteen menus by remoteness. Some previous studies have not analysed healthiness of canteen menus by school characteristics such as level of disadvantage or geographic location^(8,15,20,21)^. Other studies have analysed, with mixed findings. A cross-sectional study in New South Wales (NSW), Australia, revealed an association with schools classified as high socio-economic status having higher canteen usage compared with schools classified as low socio-economic status^(22)^. A cross-sectional study in NSW revealed no difference in nutrition by school location and level of disadvantage^(23)^, while a study in NSW revealed some variations in the availability of healthy foods and pricing and promotional strategies by school characteristics, for example, a higher proportion of schools in high socio-economic areas had healthier menu options^(24)^. Another national Australian cross-sectional study revealed an increase in proportion of schools selling less healthy snacks cheaper than healthy snacks as level of disadvantage increased^(14)^. These studies only focused on government schools^(14,15,23)^, were largely focused on NSW^(20,23–28)^ and did not include a price analysis on beverages^(14,15)^ or the presence of a healthy eating policy within the school. Moreover, as food affordability is a major determinant of the healthiness of dietary choices, it is important to determine whether school food environments support healthy choices for those with higher levels of socio-economic disadvantage (who tend to be more price sensitive)^(29)^. There is a need for studies to more comprehensively assess the factors associated with health-promoting pricing strategies in order to generalise findings more readily to a range of contexts.

Therefore, the aims of this study were to:

1. Analyse the healthiness and price of items offered by Victorian primary school canteens across various school characteristics and levels of remoteness;
2. Examine adherence to Victorian state government guidelines for provision of food and drinks in primary school canteens

## Methods

### Study design and participants

The data used in this study were from cross-sectional analyses of a convenience sample of canteen menus that were collected from primary (elementary) schools participating in three studies: the Whole of Systems Trial of Prevention Strategies for Childhood Obesity (WHO STOPS^(30)^) in 2017 (*n* 16; original study had an overall 69 % school participation rate, of which 30 % schools with menus collected in Autumn/Winter are included in this current analysis); Goulburn Valley Health Behaviours Monitoring Study in 2016 (*n* 9; original study had an overall 63 % school participation rate, of which 23 % with menus collected in Autumn/Winter are included in this current analysis)^(31)^ and Healthy Together Victoria and Childhood Obesity Study from 2016 to 2019 (*n* 23; original study had an overall 33 % school participation rate, of which 50 % schools with menus collected in Winter/Spring are included in this current analysis)^(32)^. Methods for the three studies have been previously described in detail^(30–32)^. For the Goulburn Valley Health Behaviours Monitoring Study and WHO STOPS, all primary schools in the nine local government areas were invited to participate. For the Healthy Together Victoria and Childhood Obesity Study, a strategic random sampling technique with replacement was used to invite three primary schools within twenty-six local government areas spread across metropolitan and regional Victoria. WHO STOPS^(30)^ and Healthy Together Victoria and Childhood Obesity Study^(32)^ were both childhood obesity prevention studies, focused on building community capacity to implement intervention strategies most relevant to their community context (i.e. community resources and capacity) to promote healthy eating and physical activity across a diverse range of settings. The Goulburn Valley Health Behaviours Monitoring Study was a census-style observational study aiming to understand rates of overweight/obesity, associations with health behaviours (e.g. diet and physical activity) and health-related quality of life^(31)^. Upon receiving verbal consent from the school principal to participate in the study, written information packs were mailed to the school and during school visits the school canteen menus were collected (hard copy). The presence or absence of school policies on healthy eating were also collected as dichotomous yes/no as part of the data collection and were completed by the school principals or their nominees. Note that the canteen menus examined in this study were collected from comparison sites without active intervention.

### Measures and data management

#### School characteristics and level of remoteness

For each school, collected characteristics included remoteness, sector (government or Catholic/independent), type (prep to year 6 (i.e. aged 5–12 years) or combined prep to year 12 (i.e. aged 5–18 years)), size, and socio-economic status. School characteristic data were collected from the publicly available MySchool database^(33)^, matched to the year of data collection (e.g. if menu was collected in 2016, then 2016 school characteristics were used).

Remoteness of the school were classified according to the Modified Monash Model^(34)^. The Modified Monash Model was developed based on the Australian Statistical Geography Standard and uses population numbers and road distance to classify areas into remoteness categories on a scale of MM1 to MM7, where 1 represents metropolitan locations and 7 represents very remote communities^(34)^. Classifications for each school were obtained from the publicly available ‘locator’ database provided by the Australian Department of Health^(35)^ and using school postcode.

School type was categorised into two groups for the purpose of this study: government schools and Catholic/independent schools. Whilst all Victorian schools regardless of whether they are government, Catholic or independent operate under legislative and regulatory requirements (e.g. Education and Training Reform Act 2006^(36)^ and Education and Training Reform Regulations 2017^(37,38)^), Catholic and independent schools are not part of the government system, with their own enrolment, costs, and policies^(39)^, and are not required to adhere to this state guideline^(15)^. Therefore, the school type was dichotomised to reflect any potential differences type of schools might have in adhering to the state government canteens, health eating and other food services policy^(12)^. School size classification was defined as per the Australian Education Act^(40)^: small schools (≥ 15–≤ 200 students), medium schools (> 200 to < 300 students) and large schools (≥ 300 students). The Index of Community Socio-Educational Advantage (ICSEA)^(41)^ is constructed from student characteristics (parents’ occupation and education level), the location of the school, and the proportion of Indigenous students and is designed to rank schools according to their level of socio-educational advantage. Given the average ICSEA across Australian schools is 1000, ICSEA scores were dichotomised into < 1000 or ≥ 1000, where < 1000 represents schools with lower socio-educational advantage (i.e. higher levels of disadvantage) and ≥ 1000 represents schools with higher socio-educational advantage (i.e. lower levels of disadvantage)^(41)^.

#### Menu assessment

Menu analysis was undertaken by two dietitians (AH and LA). AH undertook 100 % of the assessment with a 10 % subset analysed independently by LA to cross-check and confirm classifications (with 100 % agreement achieved). Each individual menu item was classified as ‘green’, ‘amber’, ‘red’ or ‘black’ based on its nutritional content, using the traffic light system defined in the Victorian government’s school operations policy ‘Canteens, Healthy Eating and Other Food Services’^(12)^. Examples of each classification are provided below but are not exhaustive. Examples of ‘green’ items included chicken and salad sandwich, pumpkin soup, fruit, low-fat yogurt and water. Examples of ‘amber’ items included Vegemite sandwich, ham and cheese sandwich, lasagne, popcorn, and 100 % fruit juice. Examples of ‘red’ items included meat savoury pie, hot chips (fries), salami and cheese sandwich, donut, and fruit drinks (< 100 % fruit juice). Examples of ‘black’ items included chocolate chip biscuits (cookies), confectionary, and chocolate mud cake. Traffic light classification was entered into an excel spreadsheet and exported to STATA version 15 (StataCorp)^(42)^ for analysis. The menu analysis was undertaken with the assistance of FoodChecker software provided free by the Victorian government’s Healthy Eating Advisory Service (HEAS, supported by the Victorian Division of Nutrition Australia)^(43)^ which assesses menu items against the Canteens, Healthy Eating and Other Food Services policy using the traffic light classification^(12)^. FoodChecker has a comprehensive database of products (branded, pre-made foods and drinks). A list of assumptions made during canteen menu analysis can be found in online supplementary material, Supplemental File 1. For food items not listed on FoodChecker, a similar item available on the database was used, or if the coding dietitian deemed that no item of close similarity was available, the item was added as a product. To do this, the dietitian located the product’s nutrition information panel online and added it to FoodChecker as a product or receipt (two occasions). Items were also classified into three categories: main, that is, items served as a main meal (e.g. sandwich, wrap, meat pie, herein referred to as main items), snack or beverage.

#### Food item grouping for pricing analysis

The price of individual menu items was extracted from the canteen menus to enable a detailed price analysis. There were no missing price data. The price analysis was undertaken by MB in STATA version 15 (StataCorp)^(42)^. Small hot food items such as party-pies, sausage rolls and dim sims were classified as ‘snacks’ when sold individually, or as a ‘main’ when sold in multiples of 2 or more. Food items which are unlikely to be purchased on their own, for example, sauces or optional additional sandwich toppings, were excluded from the analysis.

‘Black’ items were grouped with ‘red’ items for the purposes of price analysis. Prices of the lowest priced ‘red/black’, ‘amber’ and ‘green’ items in each menu category (main, snack and beverage) were identified. Canteens without ‘red’ items in a menu category were classified as selling their ‘green’ items cheaper in that category^(14)^. Those canteens without ‘green’ items were classified as selling ‘red’ alternatives cheaper.

### Statistical analysis

Chi-square tests were used to determine differences in the proportion of items available for purchase in each traffic light category by remoteness, sector, type, size and ICSEA with *P* < 0·05 considered a statistically significant difference. The most common ‘red’ and ‘black’ items was tabulated by ordering the highest proportion of items by each discretionary/banned food category. To examine adherence to Victorian state government guidelines for food and drink provision in primary school canteens, menu items were first tabulated by individual school into the relevant ‘green’, ‘amber’, ‘red’ and ‘black’ categories. Schools were then assessed against the School Canteens and Other School Food Services Policy which states ‘green’ items to represent > 50 % of the menu, ‘amber’ to represent < 50 % of the menu, and ‘red’ items should not be regularly available (e.g. available no more than two occasions per term) and ‘black’ items should not be supplied at any time^(12)^.

For the pricing analysis, the prices of the cheapest ‘green’, ‘amber’ and ‘red’ items in each menu category were compared using univariate linear regression to determine the mean (95 % CI) price and price difference between ‘red’ and ‘green’ alternatives. A series of univariate linear regressions were conducted for each menu category to determine the differences in price by traffic light classification. Normality of price residuals was tested using hettest command in Stata. Multi-variable analysis was not utilised to reduce the risk of a type 1 error. Note that the pricing analysis was conducted to compare items with the cheapest menu item, rather than the mean price of the menu item, as per previous methodology and the likelihood that the cheapest menu item would be the most affordable comparison for students^(14)^. Prices ($) are reported in AUD. *P*-values < 0·05 were considered significant.

Main items were further examined using exploratory univariate linear regression analysis to test whether price of cheapest main item varied by school characteristics including: size of school (small, medium and large), ICSEA score (ICSEA < 1000; ≥ 1000), school sector (government and non-government (Catholic/independent)), remoteness (MM1 (metropolitan), MM2, MM3, MM4 and MM5 (most remote in this context)) and presence of school healthy eating policy (yes and no). Multivariate linear regressions were used to explore the association between school characteristics and price and traffic light classification of menu items. An interaction term between traffic light classification and school characteristics was included in these models to explore whether the variation in the price for each traffic light category was related to school characteristic level. Due to the presence of exploratory interaction terms, *P*-values < 0·01 were considered significant to reduce the risk of type I error^(44)^.

### Sensitivity analysis

To compare results of this study to the most relevant previous study of school food prices in Australia by Billich et al.^(14)^, a sensitivity analysis was conducted. The analyses were repeated using collapsed healthiness classifications in which product traffic light classifications were grouped into ‘healthier’ (‘green’) and ‘less healthy’ (‘amber’, ‘red’ and ‘black’) items.

## Results

### School characteristics

The characteristics of participating schools are presented in Table 1. Forty-eight schools provided canteen menus offering a total of 1818 individual menu items for sale, an average of thirty-eight items per school. The estimated total enrolments was 16 146 students (prep to year 6), and the majority of schools that provided menus were government (77 %) and prep to year 6 (81 %) schools, with a relatively even representation of small, medium and large schools. The majority of schools were situated in small rural towns (MM5), and none of the schools in this sample were classified as remote or very remote communities (MM6–MM7). Most (60 %) of the schools were in communities with lower than average socio-educational advantage (ICSEA < 1000). Most schools (89 %) reported that they did have a healthy eating policy in place, and the canteen food was prepared on site in two-thirds of schools (65 %) rather than out-sourced to an external provider.

**Table 1:** School characteristics and proportion (%) of ‘green’, ‘amber’, ‘red’ and ‘black’ menu items (*n* 48 canteen menus)

School characteristic	Schools		Mean (sd) percentage menu items per school subgroup							
	*n*	%	‘Green’		‘Amber’		‘Red’		‘Black’	
			Mean	sd	Mean	sd	Mean	sd	Mean	sd
All schools combined (*n* 48)	48	100	20·8	11·0	52·6	16·7	24·7	16·8	1·9	3·9
Remoteness										
MM1 (metropolitan areas, major cities)	7	14·6	24·1	11·2	54·4	11·6	20·1	8·3	1·3	2·0
MM2 (regional centres)	3	6·2	15·0	1·0	48·3	13·6	27·3	5·5	9·3	8·5
MM3 (large rural towns)	14	29·2	22·5	12·2	46·8	17·2	29·2	22·0	1·4	2·7
MM4 (medium rural towns)	8	16·7	19·3	10·2	50·5	9·8	26·8	16·6	3·6	5·0
MM5 (small rural town)	16	33·3	19·8	11·5	58·6	20·3	21·3	16·1	0·4	1·5
	*P*-value		0·829		0·404		0·263		0·018	
Remoteness (dichotomised)										
MM1 (metropolitan areas, major cities)	7	14·6	24·1	11·2	54·4	11·6	20·1	8·3	1·3	2·0
MM2, MM3, MM4 and MM5 combined (non-metropolitan)	41	85·4	20·3	11·0	52·2	17·5	25·5	17·8	2·0	4·1
	*P*-value		0·311		0·310		0·257		0·770	
School sector										
Non-government (Catholic/independent)	11	22·9	23·5	9·3	51·2	12·7	21·4	17·3	3·8	5·8
Government	37	77·1	20·0	11·4	53·0	17·8	25·7	16·8	1·3	2·9
	*P*-value		0·133		0·834		0·586		0·142	
School type										
Primary (prep to year 6)	39	81·2	19·6	11·1	52·4	18·3	26·2	17·8	1·9	4·1
Combined (prep to year 12)	9	18·8	26·3	9·3	53·1	7·0	18·6	10·0	1·9	3·1
	*P*-value		0·057		0·724		0·669		0·595	
School size										
Small (≥15 to ≤ 200 students)	19	39·6	18·3	11·0	54·0	22·5	26·4	19·9	1·4	4·5
Medium (>200 to <300 students)	15	31·3	22·3	11·6	48·1	12·5	26·5	17·3	3·1	4·2
Large (≥300 students)	14	29·2	22·6	10·3	55·5	10·1	20·6	10·9	1·3	2·1
	*P*-value		0·265		0·722		0·492		0·318	
ICSEA										
<1000 lower advantage	29	60·4	20·2	11·4	51·0	17·0	27·3	18·3	1·6	3·2
≥1000 higher advantage	19	39·6	21·8	10·6	54·9	16·4	20·9	13·8	2·4	4·7
	*P*-value		0·462		0·508		0·690		0·670	
Written healthy eating policy*										
Yes	41	85·4	21·5	10·9	54·7	15·2	21·8	13·8	2·0	4·0
No	5	10·4	13·8	2·6	39·5	25·2	45·1	25·9	1·6	3·6
	*P*-value		0·635		0·071		0·151		0·422	
Canteen type										
In-house	31	64·6	20·4	9·3	54·2	14·5	23·1	14·2	2·4	4·4
Out-sourced	17	35·4	21·6	13·8	49·6	20·3	27·7	20·9	1·1	2·6
	*P*-value		0·636		0·553		0·515		0·583	

MM, Modified Monash Model; ICSEA, Index of Community Socio-Educational Advantage.

* Data missing for two schools; in-house – canteen on school site; out-sourced – external food retail setting provided food. *P*-value from *χ* ^2^ test. MM was used to classify remoteness^(34)^; ICSEA^(41)^: lower socio-educational advantage (i.e. higher levels of disadvantage) and higher socio-educational advantage (i.e. lower levels of disadvantage).

### Menu analysis

The percentage contribution of each traffic light category to individual school’s menu was calculated (e.g. number of items from green traffic light category/total number of items offered per school) in order to provide a comparison to national guidelines. The mean percentage contribution was then calculated for the overall sample of forty-eight schools. In this sample of forty-eight schools, canteen menus comprised of 21 % ‘green’, 53 % ‘amber’, 25 % ‘red’ and 2 % ‘black’ items (Table 1). There were no statistically significant differences in food offerings by ‘green’, ‘amber’, ‘red’ or ‘black’ classification by school sector, school size, ICSEA, presence of a policy or type of school canteen. Whilst the proportion of ‘red’, ‘amber’ and ‘green’ foods offered was similar by remoteness, there was a significant difference in the proportion of ‘black’ items by remoteness (*P* = 0·018). Schools classified as ‘regional centres’ (MM2) had the highest proportion of ‘black’ classified items (9·3 % MM2 *v*. range of 1–4 % for all other remoteness categories).

#### Healthiness of items within food categories (meal, snack and beverage)

Of the forty-eight canteen menus, all (100 %) offered mains, forty-five (94 %) offered beverages and forty (83 %) offered snacks. The 1818 menu items were classified into three food categories: meal items (*n* 1101), snack items (*n* 403) and drinks (*n* 314). ‘Green’ classified foods made up 16 % of main meal items (*n* 177), 22 % of snack items (*n* 87) and 45 % of drink items (*n* 141). ‘Amber’ classified foods dominated main (66 %, *n* 725) and snack (42 %, *n* 168) items. ‘Red’ items made up approximately one-fifth of main (18 %, *n* 198), one-quarter of snack (28 %, *n* 113) and one-third of drink (33 %, *n* 104) items. ‘Black’ items were most frequent in snacks (9 %, *n* 35). Data not shown.

In addition to examining the overall frequency (n) of green, amber, red and black items, the percentage contribution of each traffic light category for each food category (main meals, snacks and drinks) was calculated for each individual school (e.g. number of snack items from green traffic light category/total number of snack items offered per school). These results were collated to provide a mean percentage contribution representative of all schools included in the study sample.

Examination of food type (main, snack and beverage) by traffic light categories was conducted. When examining main meal items, on average 14 % were ‘green’, 64 % ‘amber’ and 22 % ‘red’. Of the forty schools that offered snack foods, 22 % were green, 39 % were amber, 32 % were red and 8 % were black. For the forty-five schools that offered drinks, 48 % were green, 19 % were amber and 33 % were red (see online supplementary material, Supplemental Table 1).

#### Examination of ‘red’ and ‘black’ items

Overall, 94 % of canteen menus (forty-five) included at least one ‘red’ or ‘black’ item regularly available. One-third of schools (33 %, sixteen schools) included banned (‘black’) items (see online supplementary material, Supplemental Table 2).

The ten most common food categories were selected for an analysis of the discretionary (‘red’ or ‘black’ classification) foods offered (Table 2). Of the 451 red or black menu items offered in the menus examined, 429 (95 %) fell into one of these ten categories. The top three most common discretionary food categories were pastry-based hot foods (37 %, e.g. pie and sausage roll), fruit-flavoured drinks (20·5 %, not including 100 % fruit juice) and cakes, muffins, sweet pastries, slices, biscuits, and bars (18·9 %).

**Table 2:** Frequency of most common ‘red’ and ‘black’ items (*n* 48 canteen menus)

Discretionary/banned food category	*n*	% of banned (Red/Black) items (*n* 429)	% of all menu items (*n* 1818)
Pastry-based hot foods	160	37·3 %	8·8 %
Fruit-flavoured drinks (not incl. 100 % fruit juice)	88	20·5 %	4·8 %
Cakes, muffins, sweet pastries, slices, biscuits and bars	81	18·9 %	4·5 %
Confectionary	23	5·4 %	1·3 %
Savoury snack foods (crisps, chips and biscuits)	21	4·9 %	1·2 %
Ice cream and ice confections	18	4·1 %	1·0 %
Deep-fried food	15	3·5 %	0·8 %
Cordial (incl. slushie)	15	3·5 %	0·8 %
High-fat processed meats	8	1·9 %	0·4 %
Total	429	100 %	23·6 %

No schools provided soft drink (sugar-sweetened carbonated beverages) for purchase.

#### Adherence to government canteen guidelines

None of the schools in this sample met the guideline of ‘everyday’ (‘green’) menu items making up the majority (more than 50 %) of the menu. Only three schools (6 %) schools met the ‘occasionally’ ‘red’ item guideline (no ‘red’ items regularly available, no ‘black’ items).

### Pricing analysis

Overall, the mean prices of all items sold in each menu category were main $3·82 (95 % CI 3·75, 3·90), beverages $2·16 (2·09, 2·23) and snacks $1·45 (1·39, 1·52) (data not shown). The mean prices of cheapest items per menu category were main $2·70 (2·55, 2·84), beverage $1·93 (1·81, 2·05) and snack $0·96 (0·86, 1·06) (Table 3). The lowest priced ‘red’ items were cheaper than the lowest priced ‘green’ items in main, beverage and snack categories (Table 3).

**Table 3:** Univariate regressions comparing price of cheapest item per food category by traffic light classification (*n* 48 canteen menus)

Menu category	Overall n items (*n*)	Mean price ($) (95 % CI)								Mean price ($) difference (95 % CI)			
		Overall	95 % CI	‘Red’	95 % CI	‘Amber’	95 % CI	‘Green’	95 % CI	‘Red’ *v*. ‘Green’	95 % CI	‘Green’ *v*. ‘Amber’	95 % CI
Main	123	2·70	2·55, 2·84	2·73	2·47, 2·98	2·29	2·06, 2·53	3·20	2·93, 3·48	−0·48	–0·85, –0·10*	−0·91	–1·27, –0·57†
Beverage	100	1·93	1·81, 2·05	1·74	1·54, 1·95	2·18	1·93, 2·43	1·94	1·76, 2·12	−0·20	–0·47, +0·08	+0·24	–0·06, +0·55
Snack	102	0·96	0·86, 1·06	1·08	0·91, 1·24	0·79	0·62, 0·95	1·07	0·87, 1·28	+0·01	–0·25, +0·27	−0·29	–0·55, –0·03‡

* *P* = 0·013.

† *P* < 0·001.

‡ *P* = 0·031.

#### Mains

Of the forty-eight schools offering mains, twenty-nine (60 %) schools offered ‘red’ mains cheaper or offered no ‘green’ mains (Table 4). Three (6 %) schools sold ‘amber’ mains only and sixteen (33 %) schools sold the lowest priced ‘green’ main item cheaper (e.g. salad) or offered no ‘red’ main items (data not shown). In all scenarios, ‘red’ item mains were cheaper than the average ‘green’ item main, when looking at lowest cost items. When looking at lowest cost items, ‘red’ mains were cheaper than ‘green’ mains (mean difference –$0·48 (95 % CI –0·85, –0·10)). Similarly, ‘amber’ mains were cheaper than green mains (–$0·91 (–1·27, –0·57)) (Table 3).

#### Beverages

Of the forty-five schools offering beverages, twenty-two (49 %) schools sold ‘red’ beverages cheaper than ‘green’ or offered no ‘green’ beverages. Twenty-three (51 %) schools sold ‘green’ beverages cheaper than ‘red’ or offered no ‘red’ beverages, when looking at lowest cost items in each category (data not shown). No other differences were found in the mean price of the cheapest items between ‘red’ and ‘green’, or ‘amber’ and ‘green’ beverage items (Table 3).

#### Snacks

Of the schools offering snacks, twenty-six (57 %) schools sold ‘red’ snacks cheaper or offered no ‘green’ snacks and four (9 %) schools sold ‘amber’ snacks only. Sixteen (35 %) schools sold ‘green’ snacks cheaper than ‘red’ or offered no ‘red’ snacks, when looking at lowest cost items (data not shown). ‘Green’ snacks were cheaper than the cheapest ‘amber’ snacks (mean difference –$0·29 (–0·55, –0·03) (Table 3). No other differences were found in the price of the cheapest items between ‘red’ and ‘green’, or ‘amber’ and ‘green’ snack items (Table 3).

#### Analysis of prices by school characteristics

Unadjusted results showed no clear pattern for association of school characteristics with whether ‘red’ main items were priced cheaper than ‘green’ except for the presence of healthy eating policy (Table 4). There were no significant differences found in price of cheapest main item by school characteristic (remoteness, school size, school sector, socio-economic advantage or reports written healthy eating policy) (data not shown). No differences were found when adjusting for traffic light classifications or when examined for interactions between school characteristic and traffic light classifications (all *P* > 0·01).

**Table 4:** Unadjusted comparison of the mean price difference between the price of the lowest priced ‘red’ or ‘amber’ main item and ‘green’ main item, by school characteristics (*n* 45 schools)*

	School total	‘Red’ cheaper than ‘Green’		Marginal price ($AUD) difference in lowest priced ‘Red’ and ‘Green’ main, compared to base socio-demographic category			School total	‘Amber’ cheaper than ‘Green’		Marginal price ($AUD) difference in lowest priced ‘Amber’ and ‘Green’ main, compared to base socio-demographic category		
School characteristic	*n*	*n* schools	%	Mean	95 % CI	*P*-value	*n*	*n* schools	%	Mean	95 % CI	*P*-value
All schools	45	29	64	−0·48	–0·85, –0·10	0·013	47	11	23	−0·91	–1·27, –0·54	*P* < 0·001
	*n*	*n* schools	%	Mean	99 % CI	*P*-value†	*n*	*n* schools	%	Mean	99 % CI	*P*-value†
Remoteness												
MM1 (metropolitan areas and major cities)	7	4	57	Base		Base	6	0	0	Base	Base	
MM2 (regional centres)	3	1	33	−0·08	–2·17, +2·01	0·921	3	0	0	−0·62	–2·74, +1·50	0·470
MM3 (large rural towns)	14	10	71	−0·37	–1·84, +1·10	0·503	14	3	21	+0·19	–1·31, +1·68	0·755
MM4 (medium rural towns)	7	6	86	−1·54	–3·20, +0·13	0·017	8	1	13	−0·39	–2·03, +1·25	0·555
MM5 (small rural town)	14	8	57	+0·35	–1·15, +1·86	0·533	16	7	44	+0·91	–0·60, +2·42	0·138
School size												
Small (≥ 15 to ≤ 200 students)	17	12	71	Base		Base	19	8	42	Base	Base	
Medium (> 200 to < 300 students)	14	8	57	−0·33	–1·55, +0·89	0·483	14	2	14	−0·60	–1·77, +0·59	0·201
Large (≥ 300 students)	14	9	64	−0·58	–1·79, +0·62	0·212	14	1	7	−1·02	–2·18, +0·15	0·029
School sector												
Government	10	5	50	Base		Base	36	10	28	Base	Base	
Non-government	35	24	69	−0·01	–1·19, +1·17	0·983	11	1	9	−0·53	–1·63, +0·57	0·228
Index of Community Socio-Educational Advantage (ICSEA)												
ICSEA < 1000 (less advantaged)	28	18	64	Base		Base	28	8	29	Base	Base	
ICSEA ≥ 1000 (more advantaged)	17	11	65	−0·20	–1·22, +0·82	0·607	19	3	16	−0·34	–1·32, +0·63	0·378
Reports written healthy eating policy												
Yes	38	23	61	Base		Base	40	8	20	Base	Base	
No	5	5	100	−0·70	–2·58, +1·18	0·337	5	3	60	+0·50	–1·37, +2·38	0·501
Missing	2	1	50	−0·67	–2·90, +1·55	0·436	2	0	0	−0·34	–2·55, +1·88	0·703

* Only 45/48 schools sold ‘red’ and/or ‘green’ main menu items. Three schools sold only ‘amber’ mains.

† *P*-value of interaction between school characteristic and traffic light classification, compared to base category.

#### Sensitivity analysis

Results were similar in the sensitivity analysis when comparing prices of ‘less healthy’ to ‘healthier’ items. ‘Less healthy’ items (‘amber’, ‘red’ and ‘black’ combined) were cheaper than the cheapest ‘healthier’ mains (mean difference –$1·07 (95 % CI –1·42, –0·72)) and snacks (–$0·31 (–0·56, –0·05)). No significant associations were found between menu category prices, or traffic light category, and school characteristics. Data not shown.

## Discussion

This study explored canteen menus in a sample of Victorian schools offering canteen services to primary school-aged children. This study has extended previous canteen analyses to Catholic/independent primary schools, analysed both food (main and snack) and beverage offerings, and examined associations not only with school characteristics such as geographic location and level of disadvantage but also the presence of a written school healthy eating policy. None of the menus analysed were fully compliant with Victorian government guidelines. Unhealthy items dominated school menus, with nine in ten schools providing ‘red’ items and one in six providing ‘black’ food items. In half of schools, ‘red’ mains, beverage and snacks were cheaper than ‘green’ items or offered no ‘green’ snacks. In this sample of canteen menus, there were no clear associations of healthiness and pricing by school characteristics except for regional centres having the highest proportion of ‘black’ items compared with all other remoteness categories examined.

### Healthiness of items offered

The current study was consistent with previous Australian studies showing that school canteen menus had poor adherence to state government guidelines^(8,14,15)^. Whilst the Victorian state government’s Department of Health provides electronic resources and training opportunities for schools^(43)^, it appears there has been little improvement in the healthiness of school canteens since an initial audit was conducted in 2008–2009^(8)^. Similar to the current study findings of 33 % menus containing black items, the 2008–2009 audit reported 37 % of 106 menus audited to contain a banned item (confectionary and high sugar drinks) and none met traffic light recommendations^(8)^. In 2012, analysis of fifty-one Victorian school canteen menus found that whilst 3 % of menus consisted of red items, only 16 % were compliant with state guidelines^(15)^. Unhealthy food offerings are also common in secondary schools with a recent national study revealing 98·5 % of 244 menus were found to contain one or more ‘red’ items and consequently did not meet canteen guidelines^(13)^.

Since 2008–2009, when an initial audit was conducted^(8)^ (note a different sample to the current study), there has been little change in the policy and programme environment to support schools to implement healthy canteen changes. Healthy canteen resources already existed^(9,11,12)^, as did a free healthy eating and physical activity programme for Victorian children attending primacy schools called ‘Kids – Go For Your Life’^(45,46)^. This programme evolved into today’s Achievement Program around 2012^(47)^ and still exists today.

One key additional resource developed during this period in 2017 was the free web-based menu planning tool delivered FoodChecker^(43)^. Despite the ability to easily check canteen menu items in this online platform, and with free support from HEAS, the healthiness of school canteens did not significantly improve.

Overall, the snacks category had the largest proportion of ‘black’ classified items (7·7 %), particularly in non-government schools (15·4 %). This was not surprising given that canteen guidelines are not mandated in non-government schools in Victoria. The most common red/black food items in this sample of schools included pastry-based hot foods, fruit-flavoured drinks (not including 100 % fruit juice), and cakes, muffins, sweet pastries, slices, biscuits and bars. In order to comply with guidelines and offer more healthy menus for students, canteens and offsite food provision services would need to remove the banned items and replace the ‘red’ ones with healthier alternatives, ideally selected in partnership with students themselves. Many major school suppliers now offer healthier versions of traditionally ‘red’ foods, such as lower fat and lower sodium meat pies, which can be classified as ‘amber’. Resources such as the Victorian Healthy Eating Advisory Service^(48)^ and the Healthy Kids Association^(49)^ provided school stakeholders (e.g. school staff, canteen managers and staff, food suppliers) a suite of resources including menu planning and promotion ideas, training and case studies to facilitate identification of healthier options.

### Pricing analysis

Approximately half of schools in this study sold ‘red’ mains, beverage and snacks cheaper or offered no ‘green’ snacks. This study found a similar price difference between ‘less healthy’ and ‘healthy’ mains (–$1·07) as in Billich et al. (–$1·00), who examined 100 primary and 100 secondary school canteen menus across Australia^(14)^. However, the current study found a smaller price difference between ‘less healthy’ and ‘healthy’ snacks (–$0·31) compared to Billich et al. (–$0·70)^(14)^. Similarly, a study by Wyse et al in 2013 of NSW primary school canteen menus found that healthier ‘main meals’ were more expensive than less healthy mains; however, less healthy items ‘drinks’, ‘snacks’ and ‘frozen snacks’ were more expensive than healthier alternatives^(20)^. Prices could certainly influence unhealthy choices if consumers are presented with cheaper and unhealthier options which save third to a fifth of the price as presented in the current study (i.e. -0·48c cheaper to buy a red compared to green main, and -0·91c cheaper to buy an amber compared to green main). Prices are known to influence choice, and increased prices for healthier food items is a missed opportunity to incentivise their purchase by children and parents^(20)^. Making the healthier option the easiest one for children and parents^(14)^ by pricing them accordingly is crucial.

### Association with school socio-demographic characteristics

In this sample of Victorian schools, there was no evidence to support associations between healthiness of menu items or pricing and school characteristics, except for regional centres (MM2) having a higher proportion of ‘black’ items. This finding is in contrast to a previous finding in NSW that higher nutritional quality canteen menus were associated with larger school sizes and in areas of high socio-economic advantage^(24)^, or a higher odds of having red items on the menu if schools were small, non-government, rurally based^(50)^. In addition to state differences, the findings may also differ between the two studies due to varying measures of socio-economic status (socio-economic indexes for areas (SEIFA)^(51)^ *v*. ICSEA) and remoteness (Accessibility/Remoteness Index of Australia^(52)^ *v*. Modified Monash Model), categorisation of school size, and differing sample size. There is an opportunity to improve the food environment, through healthier canteen menus, and subsequently the child’s diet to reduce long-term health inequities^(53)^ with mandated and monitored guidelines for all schools.

### Implications for public health

This study showed that Victorian canteen menus do not meet healthy guidelines and therefore are not providing environments which encourage healthy diets. Optimising adherence to existing policy in Victoria remains an urgent priority. Government needs to invest in strengthening enforcement of the guidelines to support schools better with healthy food provision (i.e. mandatory guidelines). Adherence of guidelines could be supported by a canteen menu monitoring system (e.g. identify low compliance, increase policy adoption, implementation and impact, and research enablers to compliance)^(15,54)^. These opportunities for action, in combination with other strategies, such as nudging – that is, small subtle changes to the physical and social environment to shift student food selection towards healthier options^(55,56)^, is vital for healthier dietary outcomes in students. Given the large proportion of time children spend at school, the current study findings and recommendations are relevant to all high-income Western countries that have school canteens on site offering food and beverages for purchase.

There has been a recent launch (January 2022) of the Victorian state-wide Vic Kids Eat Well initiative, a movement supported by the Victorian government and delivered by the Cancer Council Victoria and Nutrition Australia^(57)^. Vic Kids Eat Well focuses on transforming the food and drink environment for children with schools as a key setting^(57)^. In NSW, local-level implementation support has been found to improve adherence to government school nutrition guidelines in the majority of schools, and without having an impact on revenue^(25,58)^. Evidence suggests a multi-strategic approach strategies such as a support officer to assist with policy implementation, engagement (principals and parents), co-design and consensus with canteen managers, training, tools, resources, monitoring and feedback to schools, and marketing can improve policy implementation^(58)^. Whilst the strategies of Vic Kids Eat Well are still evolving, the addition of Healthy Kids Advisors to support local implementation along with a multi-strategic approach is optimistic. The current study findings emphasise the need for a focus in regional areas, along with a monitoring system for compliance to guidelines.

The pricing analysis in the current studies reveals opportunities for school food provision, for example, consideration of pricing policies and strategies to subsidise or reduce the cost of healthy menu items^(15)^ and disincentivising ‘amber’ products by marking them up a higher proportion^(20,59)^. Raising prices for unhealthier items can then subsidise healthier items and reduce their price^(13)^. There is evidence that pricing the healthiest main meal and snack items as the cheapest may encourage healthier choices^(14)^. Changes to the demand side, for example, school promotional strategies to increase student led demand for healthier items^(13)^, may make healthier choices easier for primary school children and more cost-effective for canteens^(13)^. Promotional strategies could include being part of a ‘meal deal’, a special price, labelled with an icon (e.g. smiley face), labelled with words to persuade purchasing and consumption such as ‘tasty’, ‘good value’ and ‘smart choice’, highlighted in an engaging way with graphic design features or colour-coded as per green traffic light system or marked as an ‘everyday’ options^(11)^. The increased demand for healthier items may allow bulk purchase and preparation of healthier foods and subsequently reduce costs^(11)^. Whilst specific to an online canteen ordering system, recent studies in NSW have revealed consumer behaviour interventions which include menu labelling, positioning, promoting, feedback and incentives can improve purchasing behaviours in primary schools^(26,28)^, an effect that has been sustained over 18 months^(27)^. Healthy canteen changes are more likely to be successful when implemented as part of a whole of school approach with engagement from principals, teachers, students, parents and the wider school community^(14)^. Future research could investigate current canteen usage to guide future planning and policy-making, in addition to qualitative data collection on the barriers to implementing and maintaining the guidelines of the government school canteen policy.

### Strengths and limitations

This study had a large number of predominantly regional and rural primary schools providing menus. This allowed us to analyse 1818 food items offered in schools and assessed this against gold standard measures of remoteness. This is also the first study to examine adherence, price and healthiness of canteen menus across varying levels of remoteness, using the Modified Monash Model^(34)^. The novelty of using this model is that classified metropolitan, regional, rural and remote areas according to geographical remoteness and town size into seven categories rather than five using Australian Statistical Geography Standard remoteness structure^(60)^. The Modified Monash Model is increasingly being used in studies to examine health-related associations and resource allocation by level of remoteness that considers socio-economic disparities within those areas^(61–64)^. A further strength of the current study is that the menu items were assessed by an experienced dietitian and cross-checked by another with 100 % agreement.

The main limitation is the use of a convenience sample – schools were not necessarily representative of Victoria, or geographic regions, or of the characteristics we were interested in such as school type, remoteness or adherence to the guidelines. However, the use of the Modified Monash Model may improve the ability to generalise to other areas^(61)^. A related limitation is that the results may be biased towards schools willing to share their menus with researchers. Schools with less healthy menus may have been more reluctant to share their menus. The canteen menus analysed represent a snapshot in time when they were collected (*n* 19 collected in 2016; *n* 12 in 2017, *n* 13 in 2018 and *n* 4 in 2019). Menus and prices may change over time or vary by season^(8,15)^ and due to canteen staff turnover and possibility that more offsite retailers/catering provision services are being utilised. This will have an impact on the generalisability of the results beyond the study sample. Future research into how offsite food provision is being utilised by school canteens is required. The cross-sectional study design was descriptive, and longitudinal studies to track adherence over time and using consistent measures of remoteness would be beneficial. The FoodChecker tool^(43)^ was utilised wherever possible for pre-packaged menu items and for recipe analysis; however, some assumptions were made during menu analysis when exact recipes were not available (see online supplementary material, Supplemental File 1). For example, dairy products, unless specified as reduced fat, were assumed to be full-fat. Similarly, sandwiches and rolls were assumed to contain margarine even if not stated on the menu, and fruit juices were classified as sweetened fruit drinks, unless identified as being 100 % natural fruit juice or by brand. Such assumptions, although made by an experienced dietitian, may have impacted the results.

## Conclusion

In this sample of forty-eight Victorian school canteen menus, there was no evidence of associations of healthiness and pricing by school characteristics except for regional centres having the highest proportion of ‘black’ (banned) items compared to all other remoteness categories examined. Furthermore, the canteen menus showed low adherence to canteen menu guidelines, with many schools still offering a high proportion of banned items. Unhealthier options were found to be less expensive than healthier options. Current Victorian state-wide canteen policies in Victoria have so far not improved the healthiness of school menus. Mandatory canteen policies need to be implemented and greater investment in establishing monitoring and additional support systems to enable healthy and affordable canteen menu items to be the easy choice for children, canteen managers and food service providers.

## Supplementary material

For supplementary material accompanying this paper visit https://doi.org/10.1017/S136898002300126X. click here to view supplementary material
