# How healthy and affordable are foods and beverages sold in school canteens? A cross-sectional study comparing menus from Victorian primary schools – ERRATUM

**DOI:** 10.1017/S1368980023002252

**Published:** 2023-12

**Authors:** Amy Hill, Miranda Blake, Laura Veronica Alston, Melanie S Nichols, Colin Bell, Penny Fraser, Ha ND Le, Claudia Strugnell, Steven Allender, Kristy A Bolton

Cambridge apologises for an error in the above article. A portion of the Results section of the abstract was duplicated during the production process.

This has been corrected in the original article.

## References

[ref1] Hill, A. , Blake, M. , Alston, L. , Nichols, M. , Bell, C. , Fraser, P. , … Bolton, K. (2023). How healthy and affordable are foods and beverages sold in school canteens? A cross-sectional study comparing menus from Victorian primary schools. Public Health Nutrition, 1-14. doi: 10.1017/S136898002300126X PMC10641611 37439210

