# Supplementary material for: How healthy and affordable are foods and beverages sold in school canteens? A cross-sectional study comparing menus from Victorian primary schools
Source: Public Health Nutr. 2023 Jul 13;26(11):2559–72. doi: 10.1017/S136898002300126X (PMC10641611; doi:10.1017/S136898002300126X)
Supplement: Supplementary file 1 [file S136898002300126Xsup001.docx]

**Supplementary Table 1: Mean proportion (%) of menu items by ‘green’, ‘amber’, ‘red’ and ‘black’ (main meal, snack or drink) and school characteristics**

|  | **Number of schools (n)** | **Meal item (%)** | | | | **Snack item (%)** | | | | **Drink item (%)** | | | | |
| --- | --- | --- | --- | --- | --- | --- | --- | --- | --- | --- | --- | --- | --- | --- |
|  |  | ‘Green’ | ‘Amber’ | ‘Red’ | ‘Black’ | ‘Green’ | ‘Amber’ | ‘Red’ | ‘Black’ | ‘Green’ | ‘Amber’ | ‘Red’ | ‘Black’ |  |
| **ALL SCHOOLS COMBINED** | 48 | 14% | 64% | 22% | 0% | 22% | 39% | 31% | 8% | 48% | 19% | 33% | 0% |  |
| **School sector** | | | | | | | | | | | | | | |
| Non-government (Catholic/Independent) | 11 | 16% | 64% | 19% | 0% | 27% | 37% | 21% | 15% | 47% | 21% | 32% | 0% |  |
| Government | 37 | 13% | 64% | 23% | 0% | 20% | 40% | 35% | 5% | 48% | 18% | 34% | 0% |  |
| *P value** |  | *0.348* | *0.591* | *0.458* | *0.064* | *0.505* | *0.650* | *0.680* | *0.197* | *0.272* | *0.507* | *0.223* | *0* |  |
| **School type** | | | | | | | | | | | | | | |
| Primary (Prep – Year 6) | 39 | 13% | 63% | 24% | 0% | 20% | 39% | 33% | 8% | 49% | 18% | 33% | 0% |  |
| Combined (Prep-Year 12) | 9 | 20% | 66% | 14% | 0% | 28% | 40% | 25% | 8% | 44% | 24% | 31% | 0% |  |
| *P value** |  | *0.27* | *0.559* | *0.759* | *0.035* | *0.143* | *0.114* | *0.478* | *0.663* | *0.694* | *0.19* | *0.633* | *0* |  |
| **School size** | | | | | | | | | | | | | | |
| Small | 19% | 10% | 66% | 25% | 0% | 18% | 44% | 33% | 6% | 55 | 10% | 35% | 0% |  |
| Medium | 15% | 17% | 58% | 26% | 0% | 20% | 34% | 34% | 12% | 41 | 25% | 34% | 0% |  |
| Large | 14% | 17% | 68% | 15% | 0% | 27% | 40% | 28% | 5% | 46 | 24% | 30% | 0% |  |
| *P value** |  | *0.251* | *0.333* | *0.374* | *0.289* | *0.686* | *0.336* | *0.53* | *0.385* | *0.192* | *0.356* | *0.408* | *0* |  |
| **Remoteness** | | | | | | | | | | | | | | |
| MM1 (metropolitan areas, major cities) | 7 | 16% | 62% | 22% | 0% | 30% | 44% | 23% | 3% | 44% | 25% | 31% | 0% |  |
| MM2 (regional centres) | 3 | 11% | 65% | 24% | 1% | 5% | 41% | 26% | 28% | 41% | 10% | 48% | 0% |  |
| MM3 (large rural towns) | 14 | 15% | 60% | 25% | 0% | 32% | 26% | 36% | 6% | 46% | 15% | 39% | 0% |  |
| MM4 (medium rural towns) | 8 | 14% | 63% | 23% | 0% | 16% | 53% | 27% | 14% | 38% | 25% | 37% | 0% |  |
| MM5 (small rural towns) | 16 | 12% | 69% | 19% | 0% | 13% | 48% | 36% | 3% | 58% | 18% | 24% | 0% |  |
| *P value** |  | *0.74* | *0.431* | *0.392* | *0.004* | *0.379* | *0.072* | *0.318* | *0.01* | *0.127* | *0.135* | *0.045* | *0* |  |
| **Remoteness (dichotomised)** | | | | | | | | | | | | | | |
| MM1 (metropolitan areas, major cities) |  | 16% | 62% | 22% | 0% | 30% | 44% | 23% | 3% | 44% | 25% | 31% | 0% |  |
| MM2, MM3, MM4, MM5 combined (non-metropolitan) |  | 14% | 64% | 22% | 0% | 20% | 38% | 33% | 9% | 49% | 18% | 33% | 0% |  |
| *P value** |  | *0.878* | *0.503* | *0.765* | *0.676* | *0.29* | *0.161* | *0.305* | *0.203* | *0.129* | *0.139* | *0.735* | *0* |  |
| **ICSEA** | | | | | | | | | | | | | | |
| <1000 | 29 | 13% | 62% | 25% | 0% | 19% | 39% | 36% | 6% | 50% | 17% | 33% | 0% |  |
| ≥ 1000 | 19 | 15% | 66% | 19% | 0% | 26% | 39% | 24% | 11% | 45% | 22% | 33% | 0% |  |
| *P value** |  | *0.472* | *0.283* | *0.66* | *0.212* | *0.174* | *0.109* | *0.376* | *0.291* | *0.110* | *0.572* | *0.262* | *0* |  |
| **Reports written healthy eating policy** | | | | | | | | | | | | | | |
| Yes | 41 | 14% | 66% | 20% | 0% | 23% | 41% | 59% | 8% | 50% | 19% | 31% | 0% |  |
| No | 5 | 6% | 52% | 42% | 0% | 3% | 32% | 54% | 11% | 42% | 47% | 41% | 0% |  |
| *P value** |  | *0.245* | *0.207* | *0.094* | *0.724* | *0.672* | *0.587* | *0.471* | *0.993* | *0.381* | *0.476* | *0.025* | *0* |  |

**analysis by chi2*
